# The complete mitochondrial genome of an Asian longicorn beetle *Dorysthenes granulosus* (Coleoptera: Cerambycidae: Prioninae)

**DOI:** 10.1080/23802359.2020.1712268

**Published:** 2020-01-16

**Authors:** Shangchao Zhou, Xialin Zheng, Wen Lu

**Affiliations:** Guangxi Key Laboratory of Agric-Environment and Agric-Products Safety, National Demonstration Center for Experimental Plant Science Education, College of Agriculture, Guangxi University, Nanning, Guangxi, China

**Keywords:** Longicorn beetle, *Dorysthenes granulosus*, Prioninae, mitochondrial genome

## Abstract

The complete mitogenome sequence of an Asian longicorn beetle *Dorysthenes granulosus* (Thomson 1860) was sequenced. The 15,858 bp long genome has the standard metazoan complement of 38 genes. These genes contain 13 protein-coding genes, 22 transfer RNA genes, 2 ribosomal RNA genes, and 1 control region. The nucleotide composition of the *D. granulosus* mitogenome was A: 39.5%, T: 31.7%, G: 10.9%, C: 17.9%. The A＋T content was 71.2%, showing strong AT skew. Phylogenetic analysis indicated that *D. granulosus* have sister relationship with *Dorysthenes paradoxus.*

## Introduction

Asian longicorn beetle *Dorysthenes granulosus* (Thomson) (Coleoptera: Cerambycidae: Prioninae), an underground pest that mainly damages sugarcane and cassava (Yu et al. 2006; Chen et al. 2012), is found in southern China, India, Myanmar and Thailand, spreading rapidly in the Chinese provinces of Hainan, Guangxi, Guangdong and Guizhou (Wickham et al. 2016). Newly hatched larvae bite the tender roots of sugarcane, and gradually eat into the stems after growing up, forming hollow sugarcane, seriously affecting the yield of sugarcane (Gong et al. 2008). Mitochondrial genome sequences are essential to a deeper understanding of the evolution of Cerambycidae and identify larva species (Wang et al. 2019). Here, the complete mitochondrial DNA (mtDNA) genome of *D. granulosus* was elucidated which has not been reported before.

In this study, specimens of *D. granulosus* were collected from the Qingxiu Mountain (22°47′N, 108°23′E) of Nanning City (Guangxi Province, China). The total genomic DNA was extracted following the modified CTAB DNA extraction protocol and stored at Guangxi Key Laboratory of Agric-Environment and Agric-Products Safety (The city of Nanning, China) with sample number of FDSW190073798. Then library was constructed and pair-end was sequenced (2*150 bp) with HiSeq (Illumina, San Diego, CA). Via SPAdes (version 3.9), approximately 15.33 G of raw data and 15.26 G of clean data were obtained for sequence assembly (Bankevich et al. 2012).

The complete mitochondrial genome of *D. granulosus* is a closed circular molecule 15,858 bp in length (GenBank accession number MN829437) and constitutive of 38 genes. These genes contain 13 protein-coding genes (PCGs), 22 transfer RNA (tRNA) genes, 2 ribosomal RNA (rRNA) genes, and 1 control region. The nucleotide composition of the *D. granulosus* mitogenome was A (39.5%), T (31.7%), G (10.9%), C (17.9%). The A＋T content was 71.2%, showing strong AT skew.

**Figure 1.**
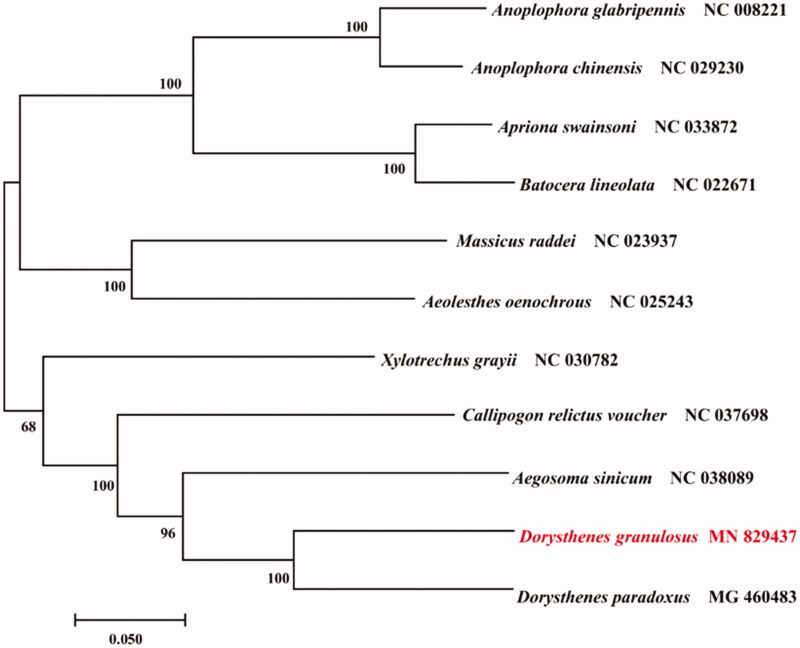
Neighbor-Joining phylogenetic tree of *Dorysthenes granulosus* and other Prioninae, Cerambycinae and Lamiinae beetles. The complete mitochondrial genome was downloaded from GenBank and the phylogenic tree was constructed by Neighbor-Joining method with 1000 bootstrap replicates were estimated using the MEGA7.0. The results showed that mtDNA of *D. granulosus* had a close relationship with that of *D. paradoxus* (Figure 1).

The phylogenetic relationship among Prioninae, Cerambycinae and Lamiinae species by Neighbor-Joining method with 1000 bootstrap replicates (Sudhir et al. 2016) were estimated using the Molecular Evolutionary Genetics Analysis Version 7.0 (MEGA7.0). The results showed that mtDNA of *D. granulosus* had a close relationship with that of *D. paradoxus* (Liu et al. 2018) (Figure 1).
